# Telehealth versus face-to-face delivery of speech language pathology services: A systematic review and meta-analysis

**DOI:** 10.1177/1357633X241272976

**Published:** 2024-10-10

**Authors:** Anna M Scott, Justin Clark, Magnolia Cardona, Tiffany Atkins, Ruwani Peiris, Hannah Greenwood, Rachel Wenke, Elizabeth Cardell, Paul Glasziou

**Affiliations:** 1 Nuffield Department of Population Health, 6396 University of Oxford, Oxford, UK; 2 Institute for Evidence-Based Healthcare, 3555 Bond University, Robina, Australia; 3 Gold Coast Health, Southport, Queensland, Australia; 4 School of Allied Health Services, 5723 Griffith University, Ipswich, Queensland, Australia

**Keywords:** Telehealth, telemedicine, speech language pathology, stuttering, Parkinson’s disease, dysphagia

## Abstract

**Background:**

There is an increasing demand for the provision of speech language pathology (SLP) services via telehealth. Therefore, we systematically reviewed randomized controlled trials comparing telehealth to face-to-face provision of SLP services.

**Methods:**

We searched Medline, Embase and Cochrane, clinical trial registries, and conducted a citation analysis to identify trials. We included randomized trials comparing similar care delivered live via telehealth (phone or video), to face-to-face. Primary outcomes included: % syllables stuttered (%SS) (for individuals who stutter); change in sound pressure levels monologue (for individuals with Parkinson's disease); and key function scores (for other areas). Where data were sufficient, mean differences were calculated.

**Results:**

Nine randomized controlled trials were included; eight evaluated video and one evaluated phone telehealth. Risk of bias was generally low or unclear, excepting blinding. There were no significant differences at any time-point up to 18 months for %SS (mean difference, MD 0.1, 95% CI −0.4 to 0.6, *p* = 0.70). For people with Parkinson's disease, there was no difference between groups in change in sound pressure levels (monologue) (MD 0.6, 95% CI −1.2 to 2.5, *p* = 0.49). Four trials investigated interventions for speech sound disorder, voice disorder and post-stroke dysphagia and aphasia; they found no differences between telehealth service delivery and face-to-face delivery.

**Conclusions:**

Evidence suggests that the telehealth provision of SLP services may be a viable alternative to their provision face-to-face, particularly to people who stutter and people with Parkinson's disease. The key limitation is the small number of randomized controlled trials, as well as evidence on the quality of life, well-being and satisfaction, and economic outcomes.

## Background

Speech Language Pathologists play a vital role in the assessment and management of a range of communication disorders (e.g., speech, language voice, fluency) and swallowing disorders across the lifespan. These disorders can markedly affect individual's quality of life, as they limit participation and social engagement,^1^ resulting in an increased risk of depression and anxiety,^2^ as well as developmental delays,^3^ and higher risk of psychological morbidity in children.^2^ However, when managed with ongoing therapy delivered by speech language pathologists, individuals with communication and swallowing disorders can achieve successful outcomes, and improved quality of life.

Due to concerns about geographic accessibility, or financial restrictions, some studies have explored the provision of speech language pathology (SLP) services by means other than their traditional in-person delivery – including telehealth (also referred to as telecare or telemedicine). Telehealth involves the provision of healthcare services remotely, synchronously (live) or asynchronously, and using a broad range of information and communications technologies, such as videoconferencing, teleconferencing, remote monitoring, mobile apps and others.^4,5^

The World Health Organization has promoted telehealth as a means of increasing accessibility, equity, quality and cost-effectiveness of health care services.^5^ Patient acceptability of telehealth is often found to be high,^6^ and patient satisfaction with consultations or treatment received via telehealth is often no different than with those received for face-to-face, across a range of conditions managed by allied healthcare providers, including PTSD,^7^ depression,^8^ anxiety,^9^ management of musculoskeletal conditions^10^ and others.^11^ Telehealth delivery of SLP, in particular, has shown client acceptability and promising clinical outcomes, for example, in vocal loudness and sentence intelligibility,^12^ and in cost savings for health services.^13^

Evidence from systematic reviews of the effectiveness of telehealth in specific population subgroups is promising. For example, a systematic review evaluating the evidence for the effectiveness of voice therapy programs in adult populations, supported the use of telehealth as a service delivery model in SLP for adults, although found that the evidence was limited in volume, most of the included studies lacked a control group, and meta-analyses were not able to be undertaken.^14^ Another review, focusing on SLP interventions delivered by telehealth to primary school-aged children with speech or language difficulties, included seven studies, showing similar improvements for children receiving interventions by telehealth and those receiving interventions in-person, similarly suggesting promising evidence in support of telehealth delivery in this group.^15^ Finally, a review of telehealth assessment or interventions for individuals with autism spectrum disorder found 14 United States-based studies utilizing a range of study designs, which suggested that telehealth delivery may be equivalent to those delivered face-to-face.^16^

As previous reviews focused on the provision of care by speech language pathologists to individuals with specific conditions or particular population subgroups, the volume and type of includable evidence was limited, and meta-analyses not possible. We therefore aimed to systematically review trials comparing the delivery of *any therapy* delivered by speech language pathologists via live telehealth (provided in real time, via videoconferencing or telephone), to its delivery face to face. We limited includable studies to randomized controlled trials only, to enable meta-analyses.

## Methods

We aimed to find, appraise and synthesize studies that compared any therapy delivered by a speech language pathologist via telehealth (video or telephone or both) to face-to-face consultations, for patients of all ages, and with any speech, language, fluency, voice or swallowing disorder. This systematic review is reported following the Preferred Reporting Items for Systematic Reviews and Meta-Analyses (PRISMA) 2020 statement. The protocol for the systematic review was developed a priori, but it was not registered with PROSPERO.

### Inclusion criteria

#### Participants

We included studies of patients of all ages with persistent primary or secondary conditions seen by a speech language pathologist (including, but not limited to, speech, language, voice, fluency and swallowing disorders).

#### Intervention and comparator

We included studies of any interventions provided by speech language pathologists in primary care settings, in any country, by telehealth (telephone or video), comparing to their provision face-to-face. Services provided in hospital settings were excluded. We included studies where the two groups received identical or nearly identical therapy in terms of intensity, dose, frequency and content, by identical or nearly identical health professionals. We excluded studies where services were provided asynchronously (e.g., store and forward transmission of patient information, mobile apps and patient monitoring devices).

#### Outcomes (primary, secondary)

The primary outcomes and secondary outcomes were identified jointly with speech language pathologists and differed by the condition addressed. For studies of individuals with stuttering, the outcomes included: % syllables stuttered (primary), and stuttering severity, time to complete treatment, satisfaction and quality of life measures (secondary outcomes). For studies of individuals with Parkinson's disease, the primary outcome was the change in sound pressure levels monologue; and the secondary outcomes included: acoustic parameters, perceptual parameters, communication partner rating, satisfaction and quality of life measures. For other studies, the outcomes included key function scores as reported in the study (primary); and time to complete treatment, satisfaction and quality of life measures (secondary outcomes).

#### Study design

We included randomized controlled trials of any design (e.g., parallel, crossover, factorial, cluster). Systematic reviews were searched for any additional includable trials. We excluded all other study designs.

### Search strategies to identify studies

This review was conducted as part of a series of systematic reviews on the effectiveness of telehealth compared to face-to-face healthcare provision in primary care or by allied healthcare providers (e.g., speech language therapists, physiotherapists and therapists) for a wide range of patient groups and conditions. Therefore, the search strings were deliberately very broad.

We searched Medline (via PubMed), Embase (via Elsevier) and Cochrane (including CENTRAL), from inception until 22 June 2023. The complete search strings for all databases are provided in an Appendix.

On 18 October 2023, we conducted a forward (citing) and backward (cited by) citation analysis using the SpiderCite tool (https://www.sr-accelerator.com/#/spidercite).

No restrictions by language or publication date were imposed. We included study reports that were published in full; publications available as abstract only (e.g., as a conference abstract) were included if they had a clinical trial registry record, or other public report, with the additional information required for inclusion. Conference abstracts only with no additional information available were excluded.

### Study selection and screening

**Figure 1.**
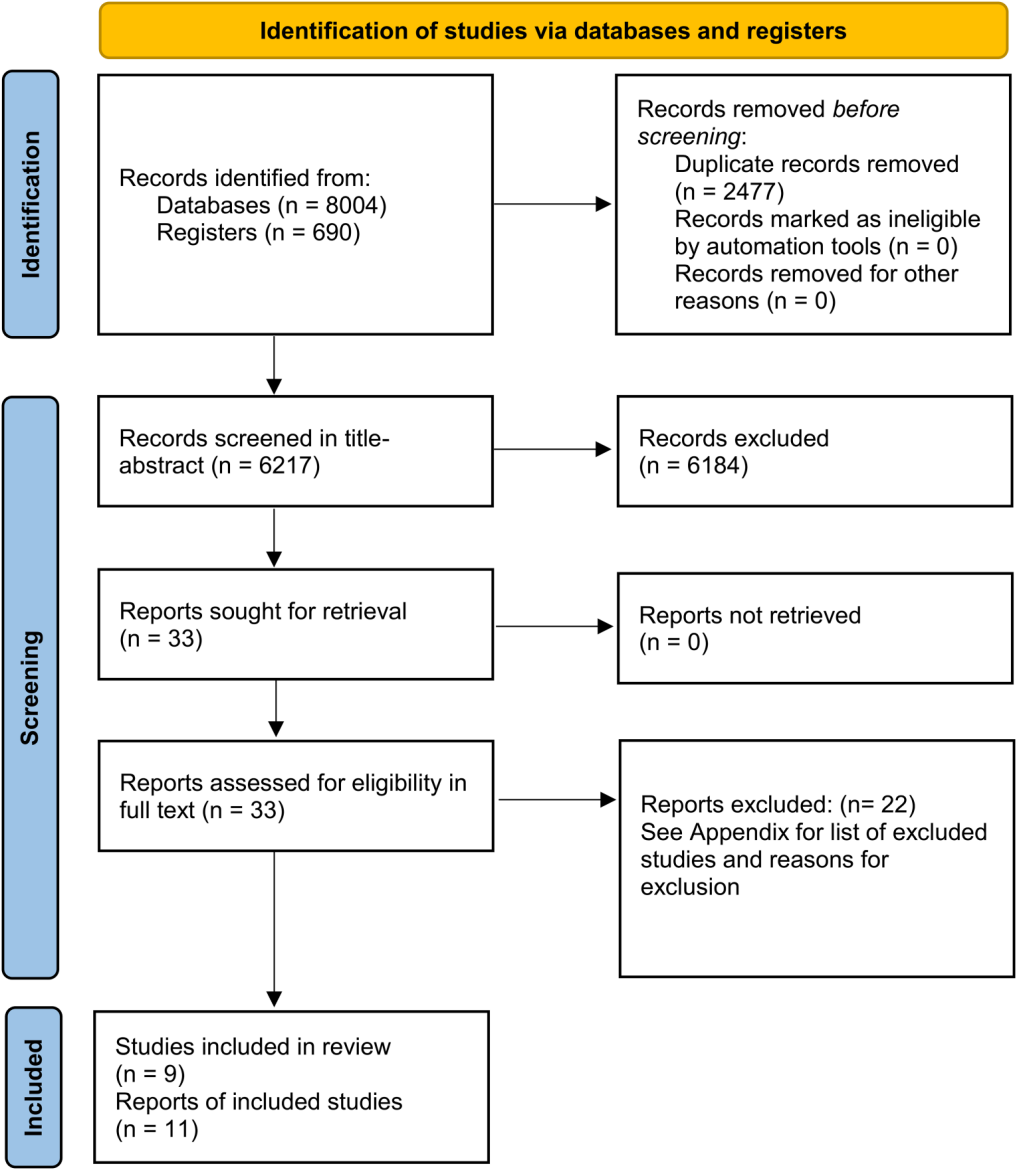
PRISMA flow diagram.

Pairs of authors (AMS, JC, MC, TA, RP, HG and PG) screened references independently, against the inclusion criteria – in title abstract and in full text. Any disagreements in decision about inclusion or exclusion were resolved by discussion or by adjudication by another author who was not a member of the original pair. Screening was conducted in either Endnote or Screenatron (https://www.sr-accelerator.com/#/screenatron), as per each screener's preference. The selection process was recorded in sufficient detail to complete a PRISMA flow diagram (see Figure 1).

### Data extraction

We used a data extraction form to extract study data, which was piloted on two studies included in this review. From each study, we extracted information on: study characteristics (methods, participants, interventions, comparators and outcomes), outcomes (primary and secondary) and data to inform the risk of bias judgements. Data were extracted by four authors independently (AMS, JC, MC and TA). Discrepancies were resolved by consensus, or reference to third author if required.

### Assessment of risk of bias in included studies

Paired review authors (AMS, JC, MC and TA) independently assessed the risk of bias for each included study using the Risk of Bias Tool 1, as outlined on the *Cochrane Handbook*.^17^ Risk of Bias Tool 1 was used in preference to Risk of Bias Tool 2, as the former enables the evaluation of potential biases due to funding or conflict of interest, under ‘other bias’ (domain 7).

Each potential source of bias was graded as low, high or unclear, supported by a quote from the relevant trial. The following domains were assessed:

1. Random sequence generation
2. Allocation concealment
3. Blinding of participants and personnel
4. Blinding of outcome assessment
5. Incomplete outcome data
6. Selective outcome reporting
7. Other bias (focusing on biases due to funding or conflict of interest).

All disagreements were resolved by discussion or by referring to a third author if required.

### Measurement of effect and data synthesis

*Review Manger 5.4* was used to calculate the treatment effect. For continuous outcomes (e.g., severity scores, satisfaction scores), we used mean difference for outcomes measured using the same scale across studies, or standardized mean difference, where outcomes were measured using different scales across studies. We undertook meta-analyses only when meaningful (i.e., when ≥2 studies or comparisons reported the same outcome).

Anticipating considerable heterogeneity, we used a random-effects model. We used the *I*^2^ statistic to measure heterogeneity among the included trials.^17^ Because we included fewer than ten trials, we did not create a funnel plot to assess the publication bias.

The individual was used as the unit of analysis, where possible. However, where data on the number of individuals with outcomes of interest was not available, we extracted the information as it was presented (e.g., mean difference in scores). We did not contact investigators or study sponsors to provide missing data.

### Subgroup and sensitivity analyses

We conducted a subgroup analysis by time-point at which the outcome was reported, but not by study age groups or condition being treated due to few included studies. We had planned to conduct a sensitivity analysis by including versus excluding studies at high risk of bias, however, due to a low number of included studies and similarity of bias profiles, we were unable to do so.

## Results

### Search results

Searches yielded in total 8694 references, resulting in 6217 references to screen in title-abstract after deduplication. We excluded 6184 references at the title-abstract screen, including 33 references for full-text screen. Twenty-two references were excluded (reasons for exclusion are provided in the Appendix), and 11 references corresponding to nine trials were included in the review (Figure 1).

### Characteristics of included studies

We included nine trials (11 references) comparing the provision of SLP services via telehealth to face-to-face.^12,18–27^ All were parallel, randomized controlled trials. Four trials took place in Australia, three trials in the United States, one in Taiwan and one in Canada. Two trials evaluated the provision of speech pathology care for individuals with stuttering, three for patients with Parkinson's disease, two for patients who were post-stroke and two for patients with other conditions. Trials ranged in size from 14 to 69 participants, and the duration of follow-up ranged from 1 week to 18 months, although four trials measured the outcomes immediately at the completion of the trial, with no further follow-up. The majority of trials compared the provision of services via video to face-to-face; one trial compared phone to face-to-face services (Table 1).

**Table 1.** Characteristics of the included studies (*n* = 9 trials).

Author Year Location	RCT design	Follow up	Number of participants randomized in total (to telehealth group, to face to face group)	Participants	Age years mean(SD)	Gender	Intervention	Telehealth modality individual session duration, frequency of sessions, programme duration.	Comparator modality individual session duration, frequency of sessions, programme duration.
***Speech language pathology trials for stuttering***									
Bridgman 2016 & Ferdinands 2019 Australia	Parallel 2-arm	18 mo.	49 (25 TH, 24 F2F)	Preschool children who stutter	NR (range 3–6)	NR	Lidcombe Program	Video 45–60 min 1x/week Programme duration NR	F2F 45–60 min 1x/week Programme duration NR
Carey 2010 Australia	Parallel 2-arm	12 mo.	40 (20 TH, 20 F2F)	Adults who stutter	NR	Female: 7/40 (18%) Male 33/40 (82%)	Camperdown Program	Phone Varied, avg. 25 sessions 30 weeks 15.5 h total	F2F Varied, avg 25 sessions 30 weeks 15.5 h total
***Speech language pathology trials for patients with Parkinson's disease***									
Constantinescu 2010 Australia	Parallel 2-arm	N/A*	34 (17 TH, 17 F2F)	Patients with Parkinson's disease	NR (range 54–85)	Female: 7/34 (21%) Male: 27/34 (79%)	LSVT^®^	Video 1hr 4x/week 4 weeks	F2F 1hr 4x/week 4 weeks
Theodoros 2016 Sayied 2020 Australia	Parallel 2-arm**	N/A*	31 (15 TH, 16 F2F)	Patients with Parkinson's disease	71 (8.8)	Female: 10/31 (32%) Male: 21/31 (68%)	LSVT^®^	Video 1hr 4x/week 4 weeks	F2F 1hr 4x/week 4 weeks
Covert 2018 USA	Parallel 2-arm	1 week	48 (18 TH, 18 F2F***)	Patients with idiopathic Parkinson's disease	NR (range 54–87)	Female:*** 2/36 (6%) Male:: 34/36 (94%)	LSVT^®^	Video 1hr 4x/week 4 weeks	F2F 1hr 4x/week 4 weeks
***Speech language pathology trials for patients post-stroke***									
Cassel 2017 USA	Parallel 2-arm	N/A*	30 (15 TH, 15 F2F)	Patients with post-stroke dysphagia	NR (>65)	Female: 18/30 (60%) Male: 12/30 (40%)	Dysphagia therapy for safe oral intake	Video 25–65 min 1x only	F2F 25–65 min 1x only
Meltzer 2018 Canada	Parallel 2-arm	12 weeks	44(22 TH, 22 F2F)	Patients with post-stroke aphasia	NR Varied by group; all mean age >60	Female: 17/44 (39%) Male: 27/44 (61%)	Communication therapy	Video 1 h 1x/week 10 weeks	F2F 1 h I x/week 10 weeks
***Speech language pathology trials for other patient groups and/or conditions***									
Grogan-Johnson 2013 USA	Parallel 2-arm	N/A*	14 (7 TH, 7 F2F)	School-aged children with speech sound impairments	NR (range 6–10)	Female: 5/14 (36%) Male: 9/14 (64%)	Speech sound intervention (personalized to each child)	Video 30 min 2x/week 5 weeks	F2F 30 min 2x/week 5 weeks
Lin 2020 Taiwan	Parallel 2-arm	8 weeks	69 (33 TH, 36 F2F)	Elderly with voice handicap index >10	NR (range 57–82)	Female: 33/69 (48%) Male: 36/69 (52%)	Voice therapy	Video 30–45 min 1x/week 8 weeks	F2F 30–45 min 1x/week 8 weeks

RCT: randomized controlled trial; mo.: month; TH: telehealth; F2F: face-to-face; LSVT: Lee Silverman Voice Treatment; NR: not reported.

*Measures reported at the completion of the trial, no longer term follow-up.

**3^rd^ arm (which was not randomized) is excluded from the present comparison.

*** Numbers randomized to each group unclear; 48 participants enrolled, 12 withdrew before finishing treatment; numbers reported are those analyzed for each group.

### Risk of bias

**Figure 2.**
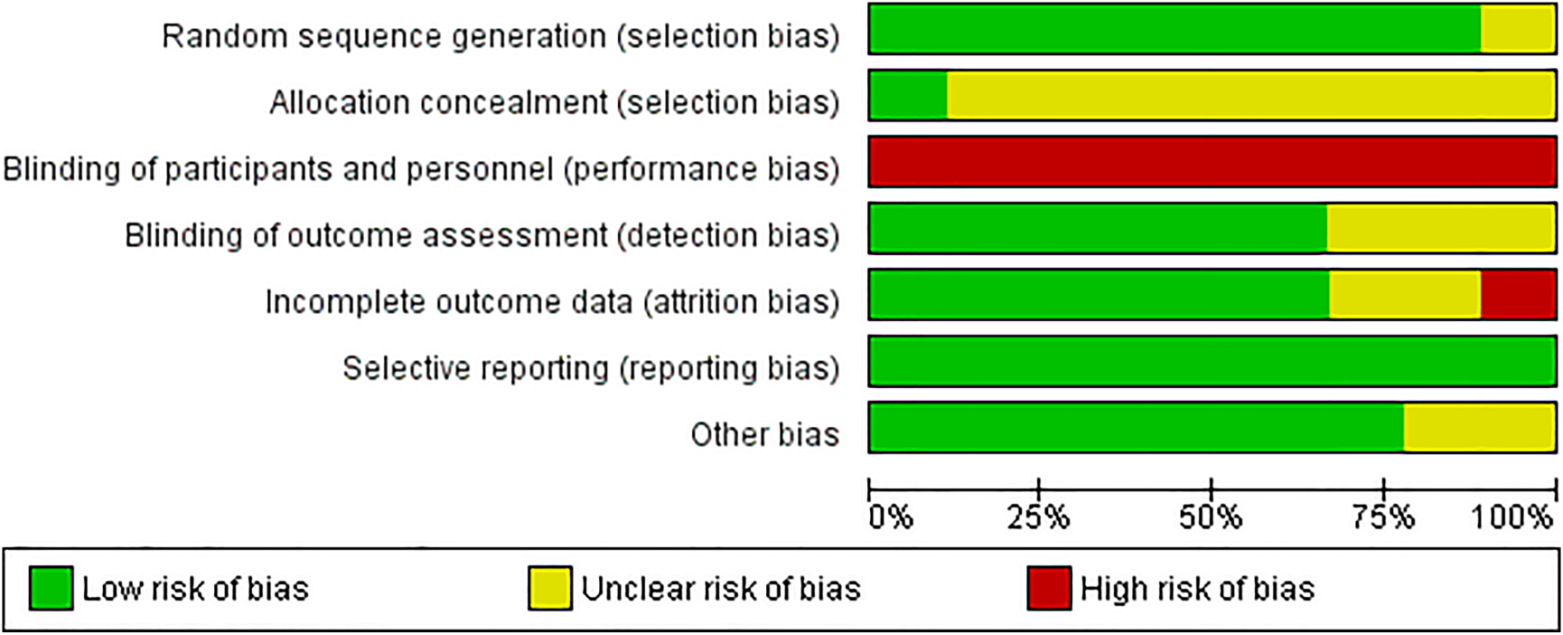
Risk of bias graph: review authors’ judgements about each risk of bias item presented as percentages across all included studies (n = 9).

Risk of bias for the included studies was generally low or unclear. Risk of bias was mostly low for random sequence generation. Most studies were rated at unclear risk of bias from allocation concealment, due to non-reporting whether concealment was used. All trials were at high risk of bias from blinding of participants and personnel, due to the nature of the compared interventions (telephone or video, versus face-to-face). Blinding of outcome assessment was mostly low or unclear – as was incomplete outcome data (although one trial was assessed at high risk). All trials were assessed at low risk of bias for selective reporting, and most were rated at low risk of bias due to other biases (assessed for potential biases due to conflicts of interest or funding sources) (Figure 2).

### Telehealth versus face-to-face SLP for patients with stuttering

Two trials^18–20^ reported on the provision of SLP services for patients with stuttering.

#### Percent syllables stuttered

**Figure 3.**
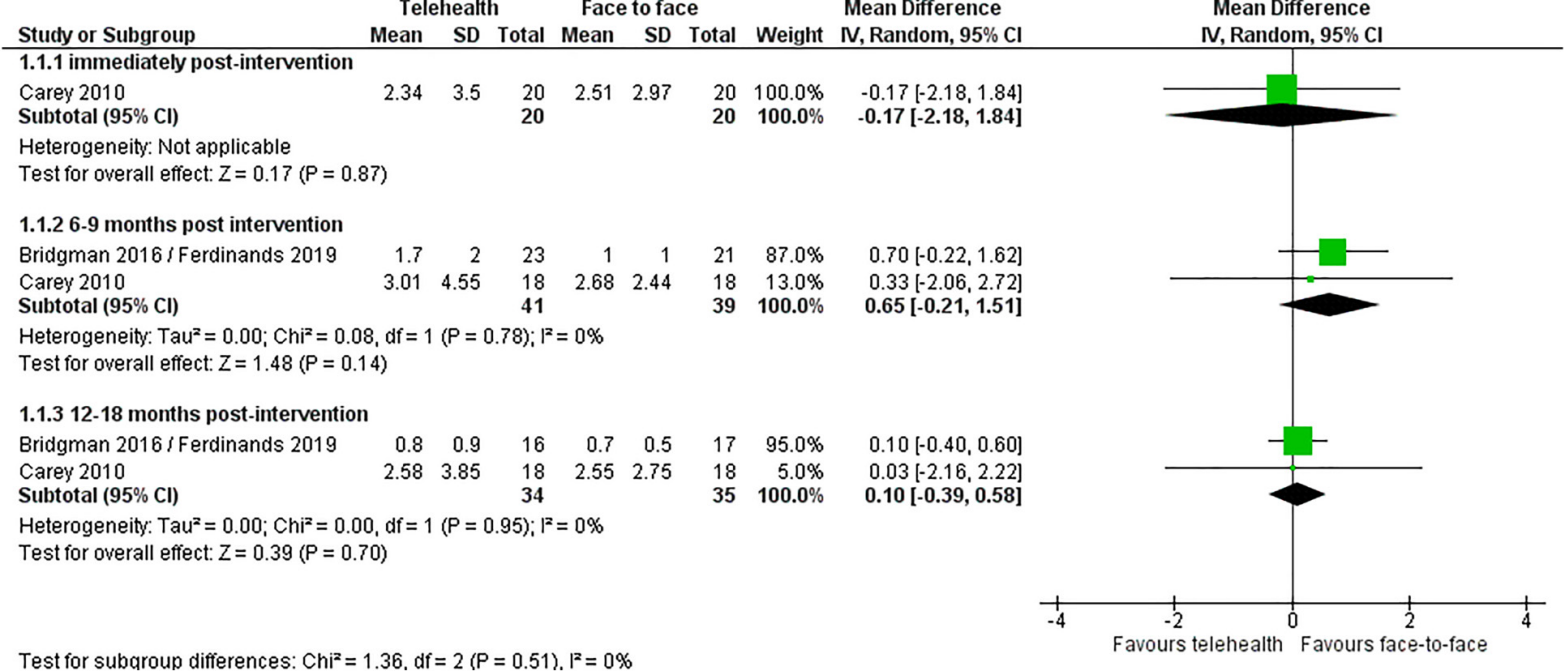
Telehealth vs. face-to-face for patients with stuttering: % syllables stuttered outcome.

For the primary outcome, percent syllables stuttered, there were no significant differences between the telehealth and the face-to-face group, immediately post-interventions (mean difference, MD −0.17, 95% CI −2.18 to 1.84), at 6–9 months post-intervention (MD 0.65, 95% CI −0.21 to 1.51), or at 18 months post-intervention (MD 0.10, 95% CI −0.39 to 0.58). Heterogeneity was very low (*I*^2 ^= 0%) (Figure 3).

#### Stuttering severity

For stuttering severity, two trials showed no differences between the telehealth and face-to-face groups. One trial with very young children^18,20^ reported no significant differences between the telehealth and face-to-face group either at nine months post-intervention (MD: 0.1, 95% CI −0.56 to 0.49, *p* = 0.88) or at 18 months post intervention (MD 0.1 95% CI −0.33 to 0.21, *p* = 0.64). The trial with adults,^19^ assessing the self-reported stuttering severity on a scale of 1–9 (none to extremely severe, respectively), also found no difference, with the mean value of 2.3 in the telehealth group and 2.4 in the face-to-face group (*p* = 0.7).

#### Time to complete treatment

Both trials reported on the time required to complete treatment. One trial found that completion of stage 1 of the Lidcombe Program required a median of 25 weeks in both the telehealth and face-to-face group. However, there was a significant difference between groups in the mean duration (minutes) of consultations: face-to-face 40.4 min (standard deviation, SD 5.2) and telehealth 33.4 min (SD 4.7), *p* < 0.001.^18,20^ Another trial found no significant difference in the mean amount of speech pathologist contact time to complete treatment (telehealth 617 min vs. face-to-face 774 min, *p* = 0.17).^19^

#### Satisfaction with treatment and/or outcomes

One trial with very young children^18,20^ measured parent satisfaction with their child's fluency. There was no significant difference between the two groups either at nine months post-intervention (*p* = 0.54) or at 18 months post-intervention (*p* = 0.74). Another trial with adults assessed treatment satisfaction, finding that participants on both groups were equally likely to describe talking on the phone as ‘extremely easy’ (*p* = 0.4); however, the telehealth treatment was significantly more frequently described as ‘extremely convenient’ (*p* = 0.018).^19^

#### Quality of life measures

Neither trial reported on quality of life measures.

### Telehealth versus face-to-face SLP for patients with Parkinson's disease

Three trials^12,21–23^ reported on the provision of SLP services for patients with Parkinson's disease. All three compared the provision of Lee Silverman Voice Treatment (LSVT) via telehealth to its provision face to face.

#### Change in sound pressure levels (monologue)

**Figure 4.**
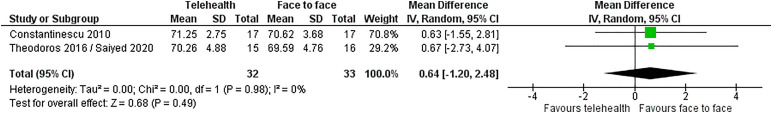
Telehealth vs. face-to-face for patients with Parkinson's disease: change in sound pressure levels (monologue).

Three trials reported on the primary outcome, change in sound pressure level (monologue); two were meta-analysable,^12,22,23^ showing no difference between the telehealth and face-to-face groups for change in sound pressure level (MD 0.64, 95% CI −1.20 to 2.48, *p* = 0.49) (Figure 4). Another trial^21^ found no difference between the two groups (telehealth mean 67.91, face-to-face mean 69.5, *p* < 0.17).

#### Acoustic parameters

**Figure 5.**
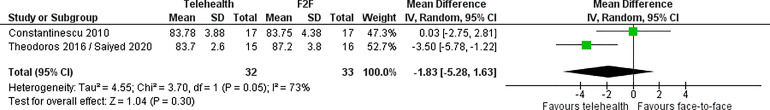
Telehealth vs. face-to-face for patients with Parkinson's disease: sustained vowel phonation SPL.

Two trials reported on sustained vowel phonation (SPL),^12,22,23^ finding no difference between groups (MD −1.83, 95% CI −5.28 to 1.63, *p* = 0.30) (Figure 5).

**Figure 6.**
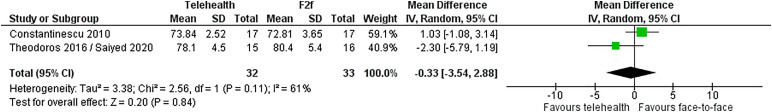
Telehealth vs. face-to-face for patients with Parkinson's disease: reading SPL.

Three trials reported on the reading SPL outcome; two were meta-analysable^12,22,23^ and showed no difference between groups (MD −0.33, 95% CI −3.54 to 2.88, *p* = 0.84) (Figure 6). One trial^21^ found a significant difference between groups on the reading passage outcome (telehealth mean 70.05 dB, face-to-face mean 72.82 dB, *p* < 0.05).

**Figure 7.**
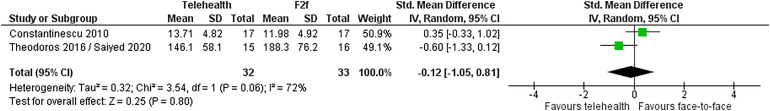
Telehealth vs. face-to-face for patients with Parkinson's disease: maximum fundamental frequency change.

Pooling two trials^12,22,23^ that reported the difference in maximum fundamental frequency range showed no difference between the telehealth and face-to-face groups (SMD −0.12, 95% CI −1.05 to 0.81, *p* = 0.80) (Figure 7).

#### Perceptual parameters

**Figure 8.**
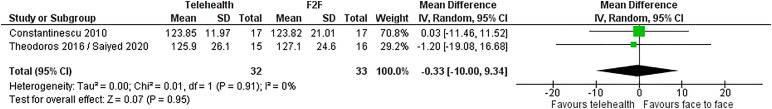
Telehealth vs. face-to-face for patients with Parkinson's disease: overall speech intelligibility.

Two trials reported on overall speech intelligibility,^12,22,23^ finding no difference between groups (MD −0.33, 95% CI −10.0 to 9.34, *p* = 0.95) (Figure 8).

**Figure 9.**
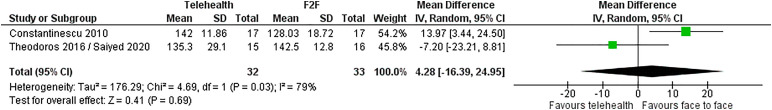
Telehealth vs. face-to-face for patients with Parkinson's disease: overall articulatory precision.

Pooling two trials that reported on overall articulatory precision^12,22,23^ showed no difference between telehealth and face-to-face groups (MD 4.28, 95% CI −16.39 to 24.95, *p* = 0.69) (Figure 9).

One trial reported on percent sentence intelligibility.^12^ The mean difference between the telehealth and face-to-face groups was not significant (MD 0.92, 95% CI −0.79 to 2.63, *p* = 0.29).

**Figure 10.**
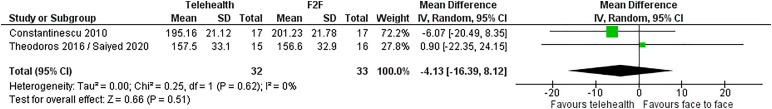
Telehealth vs. face-to-face for patients with Parkinson's disease: loudness.

Pooling two trials that reported on loudness showed no significant difference between telehealth and face-to-face groups (MD −4.13, 95% CI −16.39 to 8.12, *p* = 0.51) (Figure 10).

#### Communication partner rating

One trial^22,23^ reported on the communication partner rating, finding no difference between groups in the overall rating (MD −0.60, 95% CI −1.53 to 0.33, *p* = 0.21).

#### Quality of life/psychosocial measures

Quality of life and psychosocial measures were measured in one trial.^22,23^ There was no significant difference between the telehealth and face-to-face group in the Dysarthria Impact Profile total score (MD 4.90, 95% CI −10.38 to 20.18, *p* = 0.53), and there was no significant difference between groups in the Parkinson's Disease Questionnaire-39 Summary Index score (MD 0.30, 95% CI −7.92 to 8.52, *p* = 0.94).

#### Satisfaction

One trial^12^ reported on the participant satisfaction in the telehealth (video) delivery of the LSVT, finding that the majority of participants were very happy (47.07%) or comfortable (47.07%) participating in the sessions, and the satisfaction with the video delivery of the treatment was high, with 18% of participants reporting being satisfied, 53% more than satisfied, and 29% very satisfied.

#### Costs

One trial^22,23^ conducted an economic analysis of the cost of the one month SLP program per patient. From the health system perspective, the costs for the telehealth program were slightly higher (mean $1076, standard deviation $71) than for the face-to-face program (mean $1020, SD $0). However, from the patient perspective, the costs were considerably lower for the telehealth program (mean $247, SD $99), than for the face-to-face program (mean $831, SD $570). These differences are due to the decreased income loss (self-reported) and the absence of travel costs in the online group (an average of 438 km was travelled per person, per one-month programme, in the face-to-face group).

### Telehealth versus face-to-face SLP for patients with other conditions

Four other trials compared telehealth to face-to-face delivery of SLP services: one trial for school children with speech sound impairments,^25^ one trial for elderly persons with dysphonia,^26^ one trial for patients with post-stroke dysphagia,^24^ and one trial for patients with post-stroke aphasia.^27^

#### School children with speech sound impairments

One trial^25^ compared the delivery of speech sound intervention (personalized to each child's needs) via video versus face-to-face.

Mean Goldman-Fristoe Test of Articulation 2 scores were not significantly different between the telehealth and face-to-face groups (MD −0.06, 95% CI −0.18 to 0.06, *p* = 0.34). Mean listener judgements were also not significantly different between groups (*p* = 0.057). The mean number of sessions attended was 9.3 in the telehealth group and 9.4 in the face-to-face group (*p* = 0.73). Neither satisfaction nor quality of life outcomes were reported.

#### Voice therapy for benign voice disorders in the elderly (dysphonia)

One trial^26^ compared the delivery of voice therapy to the elderly with voice handicap index >10, via video to face-to-face.

There was no significant difference between groups post-treatment in Voice Handicap Index-10 scores, maximum phonation times, Jitter % and smoothed cepstral peak prominence. There was, however, a significant difference in shimmer (*p* = 0.04) and in noise-to-harmonic ratio (*p* = 0.01), both favouring the telehealth group (see Table 2). Neither satisfaction nor quality of life outcomes were reported.

**Table 2.** Telehealth vs. face-to-face for voice therapy for benign disorders in the elderly.

Outcome	Telehealth			Face-to-face			MD [95% CI], *p*-value	Difference between groups
	Mean	SD	*N*	Mean	SD	*N*		
VHI-10	16.8	8.94	25	13.46	9.95	24	3.34 [−1.96, 8.64], *p* = 0.22	Not significant
MPT	9.83	3.2	25	10.15	5.85	24	−0.32 [−2.98, 2.34], *p* = 0.81	Not significant
Jitter %	1.73	1.16	25	2.63	2.01	24	−0.90 [−1.82, 0.02], *p* = 0.06	Not significant
Shimmer	0.44	0.17	25	0.67	0.53	24	−0.23 [−0.45, −0.01], *p* = 0.04	Significant
NHR	0.14	0.03	25	0.19	0.09	24	−0.05 [−0.09, −0.01], *p* = 0.01	Significant
CPPs	4.39	1.3	25	4.32	1.47	24	0.07 [−0.71, 0.85], *p* = 0.86	Not significant

VHI-10: Voice Handicap Index-10; MPT: maximum phonation times; NHR: noise-to-harmonic ratio; CPPs: smoothed cepstral peak prominence.

#### Patients with post-stroke dysphagia

One trial^24^ compared the delivery of instructional methods for dysphagia via video to face-to-face, to patients with post-stroke dysphagia. The trial found no significant difference between the telehealth and the face-to-face groups in swallowing ability, with 87% of participants in the telehealth group and 80% of the participants in the face-to-face groups achieving a goal of over 80% accuracy of 15 oral intake trials (difference between groups was not significant). There was also no difference between the telehealth and face-to-face groups in the mean scores of responses with cues (*p* = 0.580) or without cues (*p* = 0.870). The trial did not report nutritional measures (e.g., blood albumin), satisfaction with the mode of treatment delivery or quality of life outcomes.

#### Patients with post-stroke aphasia

One non-inferiority trial^27^ compared communication therapy for patients with post-stroke aphasia via video versus face-to-face. There were no significant differences in reduction of aphasia severity as there was a 1.1 point average difference (90%CI: −2.05 to 4.26) in favour of telehealth compared to face-to-face. However, there was a significant difference between groups in favour of face-to-face (*p* = 0.03) for levels of self-rated communication confidence as pre-to-post gains for telehealth (*n* = 14, mean gain = 2.18, SD = 1.64) were not as high, compared to face-to-face (*n* = 14, mean gain = 4.79, SD = 3.72). However, there were no statistically significant differences between groups in functional competence, as judged by the communication partner (*p* = 0.83). Neither satisfaction nor quality of life outcomes were reported.

## Discussion

This systematic review identified nine randomised clinical trials comparing the effectiveness of SLP delivered by telehealth to conventional face-to-face delivery, for the management of persistent communication disorders in people with dysarthria following Parkinson's disease, stuttering and other conditions. Our findings indicate that SLP services delivered via telehealth provide similar outcomes compared to services delivered face to face.

The results support favourable findings from earlier systematic reviews of telehealth in SLP populations.^14,28^ Specifically, the present review confirmed that telehealth is no different to face-to-face approach for the provision of LSVT^®^ in improving speech and well-being outcomes in Parkinson's Disease, confirming the findings from earlier efficacy studies of face-to-face therapy using the LSVT^®^.^29–31^ As up to 90% of individuals with Parkinson's Disease present with dysarthria,^32^ having accessible services via telehealth may have important implications for the communication and well-being of this population.

Similarly, meta-analyses of two trials confirmed similarity of telehealth vs. face-to-face treatment for young children who stutter. Systematic reviews targeting telehealth stuttering management by Lowe et al.^33^ and McGill et al.^34^ have similarly demonstrated positive client outcomes across the lifespan with evidence for the Lidcombe Program (young children), Camperdown program (adolescents, adults) and integrated approaches (young children, school-aged children, adults). Lowe et al.^33^ noted that telehealth delivery for very young children required more clinical hours than in-clinic face-to-face treatment, however, the reported Lidcombe Program RCT^18^ did not show this. It must be noted that with the exception of the two trials reported in the current review,^18,19^ all other studies have not had the rigor of being a RCT and most have had low numbers of participants (i.e., 1–6).

We have also identified several key evidence gaps. The evidence on the quality of life outcome was reported by only one trial. This is an important gap because quality of life is a predictor of treatment success and should be routinely measured in trials.^35^ Similarly, the evidence around satisfaction with telehealth was limited – patient satisfaction was reported by only three trials, and none of the trials reported on care providers’ satisfaction. Because care providers’ satisfaction with telehealth may impact its level of adoption,^36^ this outcome likewise should be routinely measured and reported. It is worth emphasizing, however, that adoption of telehealth by care providers requires the provision of upskilling in this method of care delivery.^37^ This could be enabled, for example, by the provision of ongoing technical support service, selection of easy to use platforms, training and accreditation programmes, for example, through professional organizations or university curricula, and the provision of professional resources and guidelines around telehealth.^36^ Finally, core outcome sets are a minimum set of outcomes that could be consistently measured and reported in trials.^38^ Their routine adoption and reporting in trials of SLP interventions could both improve the quality of the evidence base and enable more robust meta-analyses and evidence-informed decision-making.

The strength of the present review is that it is the first to include only randomized controlled trials comparing live telehealth (via video or phone) to face-to-face delivery for SLP. However, this is also a limitation in that a number of observational studies that have evaluated telehealth service delivery and shown positive outcomes in populations frequently seen by speech language pathologists including aphasia, primary progressive aphasia and cognitive-communication disorders, were excluded from the review.^39–44^ On the other hand, the restriction of includable studies enabled meta-analyses to have been conducted. We also had not pre-specified the extraction of economic outcomes for the included studies. However, as one of the included studies^22,23^ conducted an economic analysis alongside the trial, we included those results. Identified studies included mostly male participants, with female participants ranging from 6% to 60% (median 32%), which somewhat limits the generalizability of the findings. However, it is worth noting that the prevalence of both Parkinson's Disease^45^ and stuttering^46^ is higher among males, and it is therefore unsurprising that the studies themselves include a higher proportion of males. The present findings are also not generalizable to speech and language therapy services provided in the hospital, or to the services provided via asynchronous telehealth, as those studies were excluded.

Despite the currently limited volume of randomized controlled trial evidence which compares live telehealth to face-to-face provision of care by speech language pathologists, the emerging picture is that telehealth is a viable service delivery option, particularly as telehealth models may in some cases increase attendance and adherence rates, and also enable speech pathology clients greater flexibility and more equitable access to services,^21^ with comparable satisfaction rates to face-to-face services.^24^

## Supplemental Material

sj-docx-1-jtt-10.1177_1357633X241272976 - Supplemental material for Telehealth versus face-to-face delivery of speech language pathology services: A systematic review and meta-analysis Supplemental material, sj-docx-1-jtt-10.1177_1357633X241272976 for Telehealth versus face-to-face delivery of speech language pathology services: A systematic review and meta-analysis by Anna M Scott, Justin Clark, Magnolia Cardona, Tiffany Atkins, Ruwani Peiris, Hannah Greenwood, Rachel Wenke, Elizabeth Cardell and Paul Glasziou in Journal of Telemedicine and Telecare
