# Supplementary material for: Telehealth versus face-to-face delivery of speech language pathology services: A systematic review and meta-analysis
Source: J Telemed Telecare. 2024 Oct 10;31(9):1203–15. doi: 10.1177/1357633X241272976 (PMC12408928; doi:10.1177/1357633X241272976)
Supplement: sj-docx-1-jtt-10.1177_1357633X241272976 - Supplemental material for Telehealth versus face-to-face delivery of speech language pathology services: A systematic review and meta-analysis [file sj-docx-1-jtt-10.1177_1357633X241272976.docx]

# Appendix 1 – Searches

**Searches run to 22/06/2023**

**RCT searches**

PubMed

("Telemedicine"[Mesh] OR "Videoconferencing"[Mesh] OR Telehealth[tiab] OR Telemedicine[tiab] OR Videoconferencing[tiab] OR ((Telephone[tiab]) AND (Consultation[tiab] OR face-to-face[tiab] OR in-person[tiab])) OR telephone-delivered[tiab])

AND

("Primary Health Care"[Mesh] OR "General Practice"[Mesh] OR rehabilitation[sh] OR "Outpatients"[Mesh] OR "Speech Therapy"[Mesh] OR Outpatient[tiab] OR “Primary health”[tiab] OR “Primary care”[tiab] OR “General practice”[tiab] OR “General practices”[tiab] OR “General practitioners”[tiab] OR “General practitioner”[tiab] OR “Family practice”[tiab] OR Physician[tiab] OR Physicians[tiab] OR Clinician[tiab] OR Clinicians[tiab] OR Therapist[tiab] OR Nurse[tiab] OR Nurses[tiab] OR Physiotherapist[tiab] OR Rehabilitation[tiab] OR Diabetes[tiab] OR Diabetic[tiab] OR Asthma[tiab] OR Depression[tiab] OR “Ïrritable bowel”[tiab] OR IBS[tiab] OR PTSD[tiab] OR “Chronic fatigue”[tiab])

AND

((Face to face[tiab]) OR “Usual care”[tiab] OR Visits[tiab] OR Visit[tiab] OR In-person[tiab] OR “In person”[tiab] OR ((Clinic[tiab] OR Centre[tiab] OR Home[tiab]) AND (Based[tiab] OR Contact[tiab])) OR Conventional[tiab] OR “Practice-based”[tiab] OR “Practice based”[tiab] OR Traditional[tiab] OR “Standard care”[tiab] OR Homecare[tiab] OR ((Routine[tiab] OR Home[tiab]) AND (Care[tiab])))

AND

("Delivery of Health Care"[Mesh] OR Delivery[tiab] OR Delivered[tiab] OR Via[tiab] OR Received[tiab])

AND

("Treatment Outcome"[Mesh] OR "Patient Satisfaction"[Mesh] OR Therapy[sh] OR Diagnosis[sh] OR “Clinical outcomes”[tiab] OR Treatment[tiab] OR Diagnostic[tiab] OR Efficacy[tiab])

AND

(Randomized controlled trial[pt] OR controlled clinical trial[pt] OR randomized[tiab] OR randomised[tiab] OR placebo[tiab] OR "drug therapy"[sh] OR randomly[tiab] OR trial[tiab] OR groups[tiab])

NOT

(Animals[Mesh] not (Animals[Mesh] and Humans[Mesh]))

NOT

(“Case Reports”[pt] OR Editorial[pt] OR Letter[pt] OR Meta-Analysis[pt] OR “Observational Study”[pt] OR “Systematic Review”[pt] OR “Case Report”[ti] OR “Case series”[ti] OR Meta-Analysis[ti] OR “Meta Analysis”[ti] OR “Systematic Review”[ti] OR “Systematic Literature Review”[ti] OR “Qualitative study”[ti] OR Protocol[ti])

CENTRAL

([mh Telemedicine] OR [mh Videoconferencing] OR Telehealth:ti,ab OR Telemedicine:ti,ab OR Videoconferencing:ti,ab OR ((Telephone:ti,ab) AND (Consultation:ti,ab OR “ face to face”:ti,ab OR “in person”:ti,ab)) OR “telephone delivered”:ti,ab)

AND

([mh "Primary Health Care"] OR [mh "General Practice"] OR [mh Outpatients] OR [mh "Speech Therapy"] OR Outpatient:ti,ab OR "Primary health":ti,ab OR "Primary care":ti,ab OR "General practice":ti,ab OR "General practices":ti,ab OR "General practitioners":ti,ab OR "General practitioner":ti,ab OR "Family practice":ti,ab OR Physician:ti,ab OR Physicians:ti,ab OR Clinician:ti,ab OR Clinicians:ti,ab OR Therapist:ti,ab OR Nurse:ti,ab OR Nurses:ti,ab OR Physiotherapist:ti,ab OR Rehabilitation:ti,ab OR Diabetes:ti,ab OR Diabetic:ti,ab OR Asthma:ti,ab OR Depression:ti,ab OR "Ïrritable bowel":ti,ab OR IBS:ti,ab OR PTSD:ti,ab OR "Chronic fatigue":ti,ab)

AND

(("Face to face":ti,ab) OR "Usual care":ti,ab OR Visits:ti,ab OR Visit:ti,ab OR "In person":ti,ab OR ((Clinic:ti,ab OR Centre:ti,ab OR Home:ti,ab) AND (Based:ti,ab OR Contact:ti,ab)) OR Conventional:ti,ab OR "Practice based":ti,ab OR Traditional:ti,ab OR "Standard care":ti,ab OR Homecare:ti,ab OR ((Routine:ti,ab OR Home:ti,ab) AND (Care:ti,ab)))

AND

([mh "Delivery of Health Care"] OR Delivery:ti,ab OR Delivered:ti,ab OR Via:ti,ab OR Received:ti,ab)

AND

([mh "Treatment Outcome"] OR [mh "Patient Satisfaction"] OR "Clinical outcomes":ti,ab OR Treatment:ti,ab OR Diagnostic:ti,ab OR Efficacy:ti,ab)

Embase

('Telemedicine'/exp OR 'Videoconferencing'/exp OR Telehealth:ti,ab OR Telemedicine:ti,ab OR Videoconferencing:ti,ab OR ((Telephone:ti,ab) AND (Consultation:ti,ab OR face-to-face:ti,ab OR in-person:ti,ab)) OR telephone-delivered:ti,ab)

AND

('Primary Health Care'/exp OR 'General Practice'/exp OR 'Outpatient'/exp OR 'Speech Therapy'/exp OR Outpatient:ti,ab OR "Primary health":ti,ab OR "Primary care":ti,ab OR "General practice":ti,ab OR "General practices":ti,ab OR "General practitioners":ti,ab OR "General practitioner":ti,ab OR "Family practice":ti,ab OR Physician:ti,ab OR Physicians:ti,ab OR Clinician:ti,ab OR Clinicians:ti,ab OR Therapist:ti,ab OR Nurse:ti,ab OR Nurses:ti,ab OR Physiotherapist:ti,ab OR Rehabilitation:ti,ab OR Diabetes:ti,ab OR Diabetic:ti,ab OR Asthma:ti,ab OR Depression:ti,ab OR "Ïrritable bowel":ti,ab OR IBS:ti,ab OR PTSD:ti,ab OR "Chronic fatigue":ti,ab)

AND

(("Face to face":ti,ab) OR "Usual care":ti,ab OR Visits:ti,ab OR Visit:ti,ab OR In-person:ti,ab OR "In person":ti,ab OR ((Clinic:ti,ab OR Centre:ti,ab OR Home:ti,ab) AND (Based:ti,ab OR Contact:ti,ab)) OR Conventional:ti,ab OR Practice-based:ti,ab OR "Practice based":ti,ab OR Traditional:ti,ab OR "Standard care":ti,ab OR Homecare:ti,ab OR ((Routine:ti,ab OR Home:ti,ab) AND (Care:ti,ab)))

AND

('health care delivery'/exp OR Delivery:ti,ab OR Delivered:ti,ab OR Via:ti,ab OR Received:ti,ab)

AND

('Treatment Outcome'/exp OR 'Patient Satisfaction'/exp OR "Clinical outcomes":ti,ab OR Treatment:ti,ab OR Diagnostic:ti,ab OR Efficacy:ti,ab)

AND

(random* OR factorial OR crossover OR placebo OR blind OR blinded OR assign OR assigned OR allocate OR allocated OR 'crossover procedure'/exp OR 'double-blind procedure'/exp OR 'randomized controlled trial'/exp OR 'single-blind procedure'/exp NOT ('animal'/exp NOT ('animal'/exp AND 'human'/exp)))

 AND [embase]/lim

**Systematic Review searches**

PubMed

("Telemedicine"[Mesh] OR "Videoconferencing"[Mesh] OR Telehealth[tiab] OR Telemedicine[tiab] OR Videoconferencing[tiab] OR ((Telephone[tiab]) AND (Consultation[tiab] OR face-to-face[tiab] OR in-person[tiab])) OR telephone-delivered[tiab])

AND

("Primary Health Care"[Mesh] OR "General Practice"[Mesh] OR rehabilitation[sh] OR "Outpatients"[Mesh] OR "Speech Therapy"[Mesh] OR Outpatient[tiab] OR “Primary health”[tiab] OR “Primary care”[tiab] OR “General practice”[tiab] OR “General practices”[tiab] OR “General practitioners”[tiab] OR “General practitioner”[tiab] OR “Family practice”[tiab] OR Physician[tiab] OR Physicians[tiab] OR Clinician[tiab] OR Clinicians[tiab] OR Therapist[tiab] OR Nurse[tiab] OR Nurses[tiab] OR Physiotherapist[tiab] OR Rehabilitation[tiab] OR Diabetes[tiab] OR Diabetic[tiab] OR Asthma[tiab] OR Depression[tiab] OR “Ïrritable bowel”[tiab] OR IBS[tiab] OR PTSD[tiab] OR “Chronic fatigue”[tiab])

AND

((Face to face[tiab]) OR “Usual care”[tiab] OR Visits[tiab] OR Visit[tiab] OR In-person[tiab] OR “In person”[tiab] OR ((Clinic[tiab] OR Centre[tiab] OR Home[tiab]) AND (Based[tiab] OR Contact[tiab])) OR Conventional[tiab] OR “Practice-based”[tiab] OR “Practice based”[tiab] OR Traditional[tiab] OR “Standard care”[tiab] OR Homecare[tiab] OR ((Routine[tiab] OR Home[tiab]) AND (Care[tiab])))

AND

("Delivery of Health Care"[Mesh] OR Delivery[tiab] OR Delivered[tiab] OR Via[tiab] OR Received[tiab])

AND

("Treatment Outcome"[Mesh] OR "Patient Satisfaction"[Mesh] OR Therapy[sh] OR Diagnosis[sh] OR “Clinical outcomes”[tiab] OR Treatment[tiab] OR Diagnostic[tiab] OR Efficacy[tiab])

AND

(Meta-Analysis[pt] OR “Systematic Review”[pt] OR Meta-Analysis[ti] OR “Meta Analysis”[ti] OR “Systematic Review”[ti] OR “Systematic Literature Review”[ti])

NOT

(“Case Reports”[pt] OR Editorial[pt] OR Letter[pt] OR “Observational Study”[pt] OR “Case Report”[ti] OR “Case series”[ti] OR “Qualitative study”[ti] OR Protocol[ti])

CDSR via the Cochrane Library

([mh Telemedicine] OR [mh Videoconferencing] OR Telehealth:ti,ab OR Telemedicine:ti,ab OR Videoconferencing:ti,ab OR ((Telephone:ti,ab) AND (Consultation:ti,ab OR “ face to face”:ti,ab OR “in person”:ti,ab)) OR “telephone delivered”:ti,ab)

AND

([mh "Primary Health Care"] OR [mh "General Practice"] OR [mh Outpatients] OR [mh "Speech Therapy"] OR Outpatient:ti,ab OR "Primary health":ti,ab OR "Primary care":ti,ab OR "General practice":ti,ab OR "General practices":ti,ab OR "General practitioners":ti,ab OR "General practitioner":ti,ab OR "Family practice":ti,ab OR Physician:ti,ab OR Physicians:ti,ab OR Clinician:ti,ab OR Clinicians:ti,ab OR Therapist:ti,ab OR Nurse:ti,ab OR Nurses:ti,ab OR Physiotherapist:ti,ab OR Rehabilitation:ti,ab OR Diabetes:ti,ab OR Diabetic:ti,ab OR Asthma:ti,ab OR Depression:ti,ab OR "Ïrritable bowel":ti,ab OR IBS:ti,ab OR PTSD:ti,ab OR "Chronic fatigue":ti,ab)

AND

(("Face to face":ti,ab) OR "Usual care":ti,ab OR Visits:ti,ab OR Visit:ti,ab OR "In person":ti,ab OR ((Clinic:ti,ab OR Centre:ti,ab OR Home:ti,ab) AND (Based:ti,ab OR Contact:ti,ab)) OR Conventional:ti,ab OR "Practice based":ti,ab OR Traditional:ti,ab OR "Standard care":ti,ab OR Homecare:ti,ab OR ((Routine:ti,ab OR Home:ti,ab) AND (Care:ti,ab)))

AND

([mh "Delivery of Health Care"] OR Delivery:ti,ab OR Delivered:ti,ab OR Via:ti,ab OR Received:ti,ab)

AND

([mh "Treatment Outcome"] OR [mh "Patient Satisfaction"] OR "Clinical outcomes":ti,ab OR Treatment:ti,ab OR Diagnostic:ti,ab OR Efficacy:ti,ab)

Embase

('Telemedicine'/exp OR 'Videoconferencing'/exp OR Telehealth:ti,ab OR Telemedicine:ti,ab OR Videoconferencing:ti,ab OR ((Telephone:ti,ab) AND (Consultation:ti,ab OR face-to-face:ti,ab OR in-person:ti,ab)) OR telephone-delivered:ti,ab)

AND

('Primary Health Care'/exp OR 'General Practice'/exp OR 'Outpatient'/exp OR 'Speech Therapy'/exp OR Outpatient:ti,ab OR "Primary health":ti,ab OR "Primary care":ti,ab OR "General practice":ti,ab OR "General practices":ti,ab OR "General practitioners":ti,ab OR "General practitioner":ti,ab OR "Family practice":ti,ab OR Physician:ti,ab OR Physicians:ti,ab OR Clinician:ti,ab OR Clinicians:ti,ab OR Therapist:ti,ab OR Nurse:ti,ab OR Nurses:ti,ab OR Physiotherapist:ti,ab OR Rehabilitation:ti,ab OR Diabetes:ti,ab OR Diabetic:ti,ab OR Asthma:ti,ab OR Depression:ti,ab OR "Ïrritable bowel":ti,ab OR IBS:ti,ab OR PTSD:ti,ab OR "Chronic fatigue":ti,ab)

AND

(("Face to face":ti,ab) OR "Usual care":ti,ab OR Visits:ti,ab OR Visit:ti,ab OR In-person:ti,ab OR "In person":ti,ab OR ((Clinic:ti,ab OR Centre:ti,ab OR Home:ti,ab) AND (Based:ti,ab OR Contact:ti,ab)) OR Conventional:ti,ab OR Practice-based:ti,ab OR "Practice based":ti,ab OR Traditional:ti,ab OR "Standard care":ti,ab OR Homecare:ti,ab OR ((Routine:ti,ab OR Home:ti,ab) AND (Care:ti,ab)))

AND

('health care delivery'/exp OR Delivery:ti,ab OR Delivered:ti,ab OR Via:ti,ab OR Received:ti,ab)

AND

('Treatment Outcome'/exp OR 'Patient Satisfaction'/exp OR "Clinical outcomes":ti,ab OR Treatment:ti,ab OR Diagnostic:ti,ab OR Efficacy:ti,ab)

AND

([cochrane review]/lim OR [systematic review]/lim OR [meta analysis]/lim OR ((Search:ti,ab OR Searched:ti,ab) AND (PubMed:ti,ab OR MEDLINE:ti,ab)) OR (Systematic:ti,ab AND Review:ti,ab) OR 'Meta analysis':ti,ab OR Meta-analysis:ti,ab OR Review:ti OR ((Systematically:ti,ab OR Reviewed:ti,ab) AND (literature:ti,ab)))

# Appendix 2 – Table of Excluded Studies

| **No.** | **Reference** | **Reason for exclusion** |
| --- | --- | --- |
| 1 | Agostini, M., M. Garzon, S. Benavides-Varela, S. De Pellegrin, G. Bencini, G. Rossi, S. Rosadoni, M. Mancuso, A. Turolla, F. Meneghello and P. Tonin (2014). "Telerehabilitation in poststroke anomia." BioMed research international **2014**: 706909-706909. | Study design |
| 2 | Aldukair, L. and D. Ward (2022). "Telepractice application for the overt stuttering assessment of children aged 6-15 years old." International journal of language & communication disorders **57**: 1050-1070. | Study design |
| 3 | Brennan DM, Georgeadis AC, Baron CR, Barker LM. The effect of videoconference-based telerehabilitation on story retelling performance by brain-injured subjects and its implications for remote speech-language therapy. Telemedicine Journal and E-Health. 2004;10(2):147-54. | Study design |
| 4 | Brignell, A., M. Krahe, M. Downes, E. Kefalianos, S. Reilly and A. Morgan (2021). "Interventions for children and adolescence who stutter: A systematic review, meta-analysis, and evidence map." Journal of Fluency Disorders **70**. | Systematic review with no further includable studies identified |
| 5 | Burns CL, Kularatna S, Ward EC, Hill AJ, Byrnes J, Kenny LM. Cost analysis of a speech pathology synchronous telepractice service for patients with head and neck cancer. Head Neck. 2017;39(12):2470-80. | Care provider (specialist) |
| 6 | Camden C, Pratte G, Fallon F, Couture M, Berbari J, Tousignant M. Diversity of practices in telerehabilitation for children with disabilities and effective intervention characteristics: results from a systematic review. Disability and Rehabilitation. 2020;42(24):3424-36. | Systematic review with no further includable studies identified |
| 7 | Campos PD, Ferrari DV. Teleaudiology: evaluation of teleconsultation efficacy for hearing aid fitting. J Soc Bras Fonoaudiol. 2012;24(4):301-8. | Care provider (specialist) |
| 8 | Cangi, M. E. and B. Toğram (2020). "Stuttering therapy through telepractice in Turkey: A mixed method study." J Fluency Disord **66**: 105793. | Study design |
| 9 | Cherney, L. (2010). "Oral Reading for Language in Aphasia (ORLA): Evaluating the Efficacy of Computer-Delivered Therapy in Chronic Nonfluent Aphasia." Topics in stroke rehabilitation **17**: 423-431. | Intervention type |
| 10 | Ciccone, N., E. Armstrong, M. Adams, D. Hersh, M. McAllister, D. Bessarab, E. Godecke, J. Coffin and M. Walley (2020). "Yarning together: Incorporating telehealth into the provision of culturally secure speech pathology services for Aboriginal Australians after brain injury." Brain Impairment **21**(SUPPL 3): 328. | Study design |
| 11 | Fridler, N., K. Rosen, M. Menahemi-Falkov, O. Herzberg, A. Lev, D. Kaplan, Y. Feldman, D. Grosberg, M. Hildesheimer and M. Shani (2012). Tele-Rehabilitation Therapy vs. Face-to-Face Therapy for Aphasic Patients. | Study design |
| 12 | Grogan-Johnson S, Alvares R, Rowan L, Creaghead N. A pilot study comparing the effectiveness of speech language therapy provided by telemedicine with conventional on-site therapy. J Telemed Telecare. 2010;16(3):134-9. | Non-comparable care in the evaluated groups |
| 13 | Grogan-Johnson S, Gabel RM, Taylor J, Rowan LE, Alvares R, Schenker J. A PILOT EXPLOR ATION OF SPEECH SOUND DISORDER INTERVENTION DELIVERED BY TELEHEALTH TO SCHOOL-AGE CHILDREN. International Journal of Telerehabilitation. 2011;3(1):31-41. | Study design |
| 14 | Hayward, K., E. Godecke, T. Russell, J. Bernhardt, R. Barker, V. Thijs, B. Campbell, S. Brownsett, G. Donnan, A. Balabanski, S. Brauer, A. Brodtmann, E. Brogan, P. Chapman, D. Copland, E. Dalton, A. Hill, T. Kleinig, A. Wong, E. Cowley, F. Ellery, B. Neibling, J. Quek and L. Churilov (2022). "PROTOCOL FOR A PHASE IIA MULTICENTRE UMBRELLA TRIAL OF INTEGRATED UPPER LIMB AND LANGUAGE IMPAIRMENT AND FUNCTIONAL TRAINING (UPLIFT) IN PEOPLE 3-24 MONTHS POST STROKE." International Journal of Stroke **17**(3): 97. | Study design (protocol only) |
| 15 | Hwang, C., C. Little, S. Russell, L. Goldberg, D. N. Kirke and M. S. Courey (2022). "Patient Satisfaction With Telemedicine Versus In-Person Speech-Language Therapy: A Systematic Review." Otolaryngology - Head and Neck Surgery **167**(1): P231. | Study design (conference abstract only) |
| 16 | Kondaurova, M., Q. Zheng, C. Donaldson, A. Betts, A. Smith and M. Fagan (2023). "The effect of telepractice on vocal turn-taking between a provider, children with cochlear implants, and caregivers: A preliminary report." Cochlear implants international **24**: 155-166. | Study design |
| 17 | Kondaurova, M., Q. Zheng, C. Donaldson and A. Smith (2023). "Effect of telepractice on pediatric cochlear implant users and provider vowel space: A preliminary report." The Journal of the Acoustical Society of America **153**: 467-479. | Study design |
| 16 | Luisa, C., K. Pawel, G. Martina, B. Francesca, F. Sara, T. Andrea and A. Michela (2021). "Telerehabilitation for people with aphasia: A systematic review and meta-analysis." Journal of Communication Disorders **92** | Systematic review (1 additional includable RCT was identified and included – Meltzer 2018) |
| 17 | Peñaloza, C., M. Scimeca, A. Gaona, E. Carpenter, N. Mukadam, T. Gray, S. Shamapant and S. Kiran (2021). "Telerehabilitation for Word Retrieval Deficits in Bilinguals With Aphasia: Effectiveness and Reliability as Compared to In-person Language Therapy." Frontiers in Neurology **12**. | Study design |
| 18 | Reverberi, C., G. Gottardo, I. Battel and E. Castagnetti (2022). "The neurogenic dysphagia management via telemedicine: a systematic review." Eur J Phys Rehabil Med **58**(2): 179-189. | Systematic review with no further includable studies identified |
| 19 | Saiyed, M., A. J. Hill, T. G. Russell, D. G. Theodoros and P. Scuffham (2022). "Cost analysis of home telerehabilitation for speech treatment in people with Parkinson's disease." J Telemed Telecare **28**(7): 524-529. | Outcomes |
| 20 | Shahouzaie, N. and M. Gholamiyan Arefi (2022). "Telehealth in speech and language therapy during the COVID-19 pandemic: a systematic review." Disabil Rehabil Assist Technol: 1-8. | Systematic review with no further includable studies identified |
| 21 | Spaccavento, S., R. Falcone, F. Cellamare, E. Picciola and R. L. Glueckauf (2021). "Effects of computer-based therapy versus therapist-mediated therapy in stroke-related aphasia: Pilot non-inferiority study." J Commun Disord **94**: 106158. | Intervention |
| 22 | Woolf, C., A. Caute, Z. Haigh, J. Galliers, S. Wilson, A. Kessie, S. Hirani, B. Hegarty and J. Marshall (2016). "A comparison of remote therapy, face to face therapy and an attention control intervention for people with aphasia: a quasi-randomised controlled feasibility study." Clin Rehabil **30**(4): 359-373. | Study design |

# Appendix 3 – PRISMA 2020 Checklist

| **Section and Topic** | **Item #** | **Checklist item** | **Location where item is reported** |
| --- | --- | --- | --- |
| **TITLE** | | |  |
| Title | 1 | Identify the report as a systematic review. | Title |
| **ABSTRACT** | | |  |
| Abstract | 2 | See the PRISMA 2020 for Abstracts checklist. | Abstract |
| **INTRODUCTION** | | |  |
| Rationale | 3 | Describe the rationale for the review in the context of existing knowledge. | Introduction section. |
| Objectives | 4 | Provide an explicit statement of the objective(s) or question(s) the review addresses. | Introduction section. |
| **METHODS** | | |  |
| Eligibility criteria | 5 | Specify the inclusion and exclusion criteria for the review and how studies were grouped for the syntheses. | Methods: inclusion criteria section. |
| Information sources | 6 | Specify all databases, registers, websites, organisations, reference lists and other sources searched or consulted to identify studies. Specify the date when each source was last searched or consulted. | Methods: search strategies section + Appendix (search strings) |
| Search strategy | 7 | Present the full search strategies for all databases, registers and websites, including any filters and limits used. | Appendix 1: Searches |
| Selection process | 8 | Specify the methods used to decide whether a study met the inclusion criteria of the review, including how many reviewers screened each record and each report retrieved, whether they worked independently, and if applicable, details of automation tools used in the process. | Methods: study selection and screening section. |
| Data collection process | 9 | Specify the methods used to collect data from reports, including how many reviewers collected data from each report, whether they worked independently, any processes for obtaining or confirming data from study investigators, and if applicable, details of automation tools used in the process. | Methods: data extraction section. |
| Data items | 10a | List and define all outcomes for which data were sought. Specify whether all results that were compatible with each outcome domain in each study were sought (e.g. for all measures, time points, analyses), and if not, the methods used to decide which results to collect. | Methods: data extraction section. |
|  | 10b | List and define all other variables for which data were sought (e.g. participant and intervention characteristics, funding sources). Describe any assumptions made about any missing or unclear information. | Methods: data extraction section. |
| Study risk of bias assessment | 11 | Specify the methods used to assess risk of bias in the included studies, including details of the tool(s) used, how many reviewers assessed each study and whether they worked independently, and if applicable, details of automation tools used in the process. | Methods: Assessment of the risk of bias section. |
| Effect measures | 12 | Specify for each outcome the effect measure(s) (e.g. risk ratio, mean difference) used in the synthesis or presentation of results. | Methods: Measurement of effect and data synthesis section |
| Synthesis methods | 13a | Describe the processes used to decide which studies were eligible for each synthesis (e.g. tabulating the study intervention characteristics and comparing against the planned groups for each synthesis (item #5)). | Methods: Measurement of effect and data synthesis section |
|  | 13b | Describe any methods required to prepare the data for presentation or synthesis, such as handling of missing summary statistics, or data conversions. | Methods: Measurement of effect and data synthesis section |
|  | 13c | Describe any methods used to tabulate or visually display results of individual studies and syntheses. | Methods: Measurement of effect and data synthesis section |
|  | 13d | Describe any methods used to synthesize results and provide a rationale for the choice(s). If meta-analysis was performed, describe the model(s), method(s) to identify the presence and extent of statistical heterogeneity, and software package(s) used. | Methods: Measurement of effect and data synthesis section |
|  | 13e | Describe any methods used to explore possible causes of heterogeneity among study results (e.g. subgroup analysis, meta-regression). | Methods: Measurement of effect and data synthesis section |
|  | 13f | Describe any sensitivity analyses conducted to assess robustness of the synthesized results. | Methods: Measurement of effect and data synthesis section |
| Reporting bias assessment | 14 | Describe any methods used to assess risk of bias due to missing results in a synthesis (arising from reporting biases). | Not applicable. |
| Certainty assessment | 15 | Describe any methods used to assess certainty (or confidence) in the body of evidence for an outcome. | Not applicable. |
| **RESULTS** | | |  |
| Study selection | 16a | Describe the results of the search and selection process, from the number of records identified in the search to the number of studies included in the review, ideally using a flow diagram. | Results: search results section |
|  | 16b | Cite studies that might appear to meet the inclusion criteria, but which were excluded, and explain why they were excluded. | Appendix B |
| Study characteristics | 17 | Cite each included study and present its characteristics. | Results: summary of included trials |
| Risk of bias in studies | 18 | Present assessments of risk of bias for each included study. | Results: risk of bias section |
| Results of individual studies | 19 | For all outcomes, present, for each study: (a) summary statistics for each group (where appropriate) and (b) an effect estimate and its precision (e.g. confidence/credible interval), ideally using structured tables or plots. | Results: TH vs F2F SLP for patients with stuttering section  Results: TH vs F2F SLP for patients with Parkinson’s disease section |
| Results of syntheses | 20a | For each synthesis, briefly summarise the characteristics and risk of bias among contributing studies. | Results: TH vs F2F SLP for patients with stuttering section  Results: TH vs F2F SLP for patients with Parkinson’s disease section |
|  | 20b | Present results of all statistical syntheses conducted. If meta-analysis was done, present for each the summary estimate and its precision (e.g. confidence/credible interval) and measures of statistical heterogeneity. If comparing groups, describe the direction of the effect. | Results: TH vs F2F SLP for patients with stuttering section  Results: TH vs F2F SLP for patients with Parkinson’s disease section |
|  | 20c | Present results of all investigations of possible causes of heterogeneity among study results. | Results: TH vs F2F SLP for patients with stuttering section  Results: TH vs F2F SLP for patients with Parkinson’s disease section |
|  | 20d | Present results of all sensitivity analyses conducted to assess the robustness of the synthesized results. | No sensitivity analyses conducted. |
| Reporting biases | 21 | Present assessments of risk of bias due to missing results (arising from reporting biases) for each synthesis assessed. | Not applicable |
| Certainty of evidence | 22 | Present assessments of certainty (or confidence) in the body of evidence for each outcome assessed. | Not applicable |
| **DISCUSSION** | | |  |
| Discussion | 23a | Provide a general interpretation of the results in the context of other evidence. | Discussion section |
|  | 23b | Discuss any limitations of the evidence included in the review. | Discussion section |
|  | 23c | Discuss any limitations of the review processes used. | Discussion section |
|  | 23d | Discuss implications of the results for practice, policy, and future research. | Discussion section |
| **OTHER INFORMATION** | | |  |
| Registration and protocol | 24a | Provide registration information for the review, including register name and registration number, or state that the review was not registered. | Protocol for the overall review was developed a priori but not registered. |
|  | 24b | Indicate where the review protocol can be accessed, or state that a protocol was not prepared. | From study authors. |
|  | 24c | Describe and explain any amendments to information provided at registration or in the protocol. | Reported in the relevant methods section. |
| Support | 25 | Describe sources of financial or non-financial support for the review, and the role of the funders or sponsors in the review. | Funding and COI statement (Appendix) |
| Competing interests | 26 | Declare any competing interests of review authors. | Funding and COI statement (Appendix) |
| Availability of data, code and other materials | 27 | Report which of the following are publicly available and where they can be found: template data collection forms; data extracted from included studies; data used for all analyses; analytic code; any other materials used in the review. | From study authors. |

# Appendix 4 – Funding and COI statements

**COI and funding disclosures**

This systematic review was commissioned by the Department of Health and Aged Care, Canberra, Australia, as part of a series of systematic reviews on the effectiveness of telehealth within primary care in 2020-21 and their update in 2023. The funder was involved in establishing the parameters of the study question (PICO). The funder was not involved in the conduct, analysis, or interpretation of the systematic review, or in the decision to submit the manuscript for publication.

The authors report no other actual or potential conflicts of interest.
